# Social Entrepreneurship: A Case Study From Brazil

**DOI:** 10.9745/GHSP-D-15-00182

**Published:** 2016-03-25

**Authors:** Phil Harvey

**Affiliations:** a DKT International, Washington, DC, USA

## Abstract

Through careful sourcing of commodities, cost-cutting efficiencies, and realistic pricing, 3 large contraceptive social marketing programs evolved into profit-making enterprises while continuing to make low-priced contraceptives available to low-income consumers on a substantial scale.

Philanthropic and humanitarian organizations are increasingly turning to business models to achieve their social objectives. “Market-based approaches,” says the Acumen Fund, “have the potential to grow after charitable dollars run out, and they must be a part of the solution to the big problem of poverty.”^1^ Virginia-based Ashoka seeks to achieve social objectives by investing in individual entrepreneurs and “changemakers” in developing countries. An example is Fábio Rosa, an Ashoka fellow, who helped bring electricity to large parts of rural Brazil, cutting rural electrification costs substantially in the process.^2^

These organizations and many others have recognized that private business models hold important lessons for achieving social objectives and that, in the right circumstances, the profit motive can be harnessed to reduce poverty and advance human well-being. Thus, more and more nonprofits are looking to market-based techniques—techniques used by profitable businesses—to tackle nonprofit objectives.

Private business models hold important lessons for achieving social objectives.

DKT International is a nonprofit family planning organization I founded in 1989, now directed by Chris Purdy. DKT has taken an unusual approach to mixing social objectives with profitability. It hires managers in new locations, or sends expatriate managers to such locations, to start projects in the social marketing of contraceptives and, where rising incomes allow it, DKT grows those projects into profitable local enterprises that become permanent parts of the local commercial community.

This pattern has emerged only recently. When DKT began operations in 1989, most of the countries where it operated were too poor for project managers to consider profit potential, and that wasn’t the fundamental purpose of these programs anyway. DKT’s objective was to provide affordable contraceptives to the poor. We assumed that the contraceptives we sold through a system called social marketing would have to be subsidized indefinitely. However, this has changed over time due to the confluence of several factors:

- The economic development of low-income countries and the resulting increase in consumer income, especially in Latin America and Asia
- The declining relative prices of contraceptives due to competition among many new Asian pharmaceutical, IUD, and condom manufacturers
- The increased size of DKT’s programs, which has made it possible to achieve economies of scale

Thus, it has been possible for DKT to move several of its projects toward financial self-sufficiency and, in 3 particular country programs (Brazil, Indonesia, and the Philippines), to generate significant profits that are used to subsidize DKT’s programs in less affluent countries. Using the DKT program in Brazil as an example, this paper describes a new design for bringing family planning social marketing programs to financial self-sufficiency and outlines the steps needed to generate substantial program revenue while maintaining a close check on affordability of a program's contraceptives to local consumers.

## SOCIAL MARKETING AND AFFORDABILITY

Social marketing itself follows a commercial design. Originally conceived and developed in 1967 in India,^3^ contraceptive social marketing programs are designed to make low-cost, attractively packaged contraceptives (originally condoms and oral contraceptives; now including virtually all methods) as easily available in towns and remote villages as tea, cigarettes, or Coca Cola. The design concept is simple: with the aid of market research, a program manager creates new contraceptive brands (or, occasionally, adapts existing brands), orders a start-up inventory, hires a distribution company or companies to get the product into existing commercial channels, and backs the sales and distribution effort with intensive advertising in mass media (e.g., “Until You Want Another Child, Rely on Preethi Condoms,” a successful slogan used in Sri Lanka) and point-of-purchase retail displays, posters, event sponsorships, T-shirts, and other promotions. The approach has worked remarkably well. In 2014, there were 82 social marketing programs operating in 62 developing countries serving just under 70 million couples,^4^ which amounts to about 18% of all married couples using modern birth control in the developing world (China excluded).^5^^,^^6^

In 2014, there were 82 contraceptive social marketing programs operating in 62 developing countries.

While financial self-sufficiency—or profitability—was never part of the original design of these programs, most managers of donor-supported development efforts are intrigued by the prospect of their own programs becoming self-financing. Revenue earned in the marketplace means less dependence on donors, a liberating prospect that means fewer proposals and reports to write and a psychological sense of independence and achievement. Thus, as incomes gradually moved up in Asia and Latin America, DKT’s managers there began analyzing their contraceptive costs and their selling prices to see whether it might be possible, as a first step, to recover their contraceptive costs—cost of goods sold—by charging prices that were high enough to achieve this while still maintaining consumer prices within affordability guidelines.

The core guideline is, for contraceptives to be within reach of low-income consumers, a year’s supply of contraceptives should cost a couple no more than 0.25% of annual per-capita gross national income (GNI) adjusted for purchasing power parity (PPP). Other yardsticks such as the price of a cup of tea, a single cigarette, or low-cost analgesics are also considered. The formula has been tested repeatedly in DKT’s programs, and in some of Population Service International’s (PSI’s) programs, and appears to be a good yardstick, one that maximizes volume. A paper analyzing condom prices and sales volumes in social marketing programs in 1994 concluded that reasonably low prices were required for high sales volumes,^7^ and those conclusions were subsequently tested in DKT’s programs. The 0.25% guideline resulted. The standard means that, in a country like Brazil with a PPP-adjusted per-capita GNI of US$14,800 (2013), a year’s supply of contraceptives should cost no more than $37 and, allowing 100 condoms for a year’s supply,* a single condom should cost no more than 37¢. In Indonesia, the lowest-priced condom must cost no more than 22¢ and the least expensive pill less than $1.73 (allowing for 13 cycles). In Ethiopia, DKT customers pay only a bit more than 2¢ (in local currency) for a condom; thus, no profit margin is possible.

For contraceptives to remain affordable for low-income consumers, a year’s supply of contraceptives should cost a couple no more than 0.25% of annual per-capita GNI.

## DKT PROGRAM IN BRAZIL

The Brazil project was one of DKT’s first contraceptive social marketing endeavors. It was incorporated there in 1990 as DKT do Brasil (DKT/B) (www.dkt.com.br), a commercial company wholly owned by DKT International in Washington, DC. At the time, 2 Brazilian condom manufacturers—the Brazilian arm of Johnson & Johnson (J&J) and Inal Ltda.—shared what was essentially an oligopoly there (J&J’s Jontex brand in particular was dominant). Their locally manufactured condoms typically sold at retail for 60¢ to more than $1 in equivalent local currency.

As a new company, DKT/B’s first task was to break the import barrier in order to bring its brands to the market at much lower prices. Fortunately, Brazil was beginning to liberalize its economic and trade policies and DKT/B was allowed to import condoms on a significant scale. Good-quality, well-packaged Asian condoms were available for around 3¢ each. Even with transport and import tariffs, this made it possible to set the consumer price at about 15¢, which meant from the outset a dramatically lower price for consumers yet still with a small margin on each sale (the all-in cost was between 4¢ and 5¢; the project sold to the trade for 6¢ to 7¢).

Sales of DKT/B “Prudence” condoms got underway in 1991, and 400,000 were sold that year (Table 1). In 1992 the total sold was 3 million. By 1999, DKT/B was selling more than 40 million Prudence condoms annually, and the number was growing.

By 1999, DKT’s Brazil project was selling more than 40 million condoms each year.

**TABLE 1:** Commercial Sales and Government Distribution of Male Condoms, Brazil, 1991–2014

Year	Volume (million pieces)			Population (millions)	Volume per Capita	DKT (million pieces)	DKT Commercial Market Share (%)
	Commercial	Government	Total				
1991	82	10	**92**	152	0.608	0.41	0.49%
1992	97	10	**107**	155	0.694	3.08	3.17%
1993	116	10	**126**	157	0.801	6.76	5.83%
1994	139	16	**155**	159	0.972	11.57	8.32%
1995	169	16	**185**	162	1.143	18.39	10.88%
1996	207	16	**223**	164	1.356	26.89	12.99%
1997	255	16	**271**	167	1.624	33.60	13.18%
1998	300	39	**339**	170	2.000	41.38	13.79%
1999	310	39	**349**	172	2.029	42.12	13.59%
2000	370	77	**447**	175	2.562	55.79	15.08%
2001	380	150	**530**	177	2.994	64.09	16.87%
2002	390	200	**590**	179	3.289	62.99	16.15%
2003	395	300	**695**	182	3.823	50.68	12.83%
2004	400	154	**554**	184	3.011	58.61	14.65%
2005	410	202	**612**	186	3.289	66.25	16.16%
2006	422	230	**652**	188	3.468	69.14	16.37%
2007	427	119	**546**	190	2.871	78.48	18.40%
2008	435	406	**841**	192	4.385	82.51	18.97%
2009	438	465	**903**	194	4.667	76.02	17.36%
2010	450	327	**777**	195	3.981	83.26	18.50%
2011	452	496	**948**	197	4.813	101.51	22.46%
2012	470	338	**808**	199	4.064	112.04	23.84%
2013	475	610	**1,085**	200	5.415	113.13	23.83%
2014	470	431	**901**	202	4.457	102.06	21.72%

Source: DKT do Brasil and Brazilian government records.

DKT’s head office investment had been substantial. Between 1990 and 1995, DKT invested $3.3 million of its own private funds in Brazil; this investment was made cheerfully because the project was succeeding. During that same period, it had achieved 402,000 couple-years of protection (CYPs) for a cost of $8.20 per CYP. (One CYP is the provision of contraceptive products or services sufficient to protect 1 couple from pregnancy for 1 year.) The$8 cost was considered highly cost-effective at the time (DKT’s standards have since become more rigorous), and the project was judged to be both substantial and efficient. Condom manufacturers in Brazil had begun lowering their prices in the face of our low-priced competition and we took considerable satisfaction from having increased the availability of contraception and protection from sexually transmitted infections in Latin America’s most populous country. (HIV/AIDS had become a significant threat in Brazil by then.)

And the market was growing fast. In 1991, Brazil’s commercial condom market was 82 million pieces. By 2000, it had reached 370 million pieces (Table 1). Could we take credit for that? Some of it, unquestionably, was due to our role in lowering consumer prices for condoms; other parties were now importing too, and competition was heating up. On the other hand, the Brazilian government deserves much credit for the surge in overall condom sales. During this period, the government had been heavily promoting condom use for HIV prevention with highly creative and attention-getting ads on TV, radio, and other media. They had also begun giving away free condoms, which stimulated interest in the product. Other private organizations also played a part. A complete statistical summary of DKT sales, total commercial sales, and government distribution is provided in Table 1.

DKT/B’s high volume of condom sales attracted the attention of international donors, including the United States Agency for International Development (USAID). After proposal writing and negotiations, an agreement was reached and USAID invested $4.8 million in the project during 1997–2003. A private foundation also provided some support toward the end of this period.

This presented an interesting dilemma. While program managers were pleased to have the donors’ funds, which made it possible to extend our reach with HIV prevention communication (and helped pay some operating overheads), the nature of the Brazilian organization began to change in ways that we did not find completely satisfactory. We had seen by then that DKT/B had the potential to become entirely self-sustaining—or even profitable—if it was properly managed. The introduction of outside donor funds required the project to take on a great many tasks that were not part of the profit-making model and, while most of those activities were valuable, they represented a considerable distraction for management, drawing attention away from the marketing and selling functions. Much of the company’s effort during this period, for example, required working with local nongovernmental organizations on HIV/AIDS communication, only some of which involved condoms and all of which involved a great deal of administrative work.

In September of 2003, USAID abruptly ended its support for the program and informed DKT that funding would not be renewed. The unexpected loss of these funds was a shock, but we also recognized an opportunity. Project managers quickly streamlined the operation, reducing staff from 28 to 15, consolidating the office into a smaller space, selling some equipment, and cutting back on generic advertising. Thus began the metamorphosis of DKT/B into a lean and efficient business—a business that we were convinced could achieve full profitability and still fulfill its mission of making affordable contraceptives conveniently available to Brazilian couples. (Profitability here means coverage of all costs, including overheads and taxes, with a net profit remaining.)

## CROSS-SUBSIDIZING: A WAY TO BE BOTH PROFITABLE AND AFFORDABLE

One way of achieving profitability without sacrificing affordability is cross-subsidization. This involves the introduction of new brands and brand variants at higher prices while still maintaining at least 1 brand that meets the affordability guidelines. For DKT do Brasil, this meant offering more and different condom brands, sold at higher prices. In 2000, for example, just 24% of DKT’s Brazilian sales revenue came from premium condom products sold at higher prices; by 2011 that percentage had jumped to 68%. This has required considerable ingenuity. DKT’s offerings in Brazil include condoms with colors and aromas (strawberry, chocolate, mint, tutti-frutti, banana, cola, and watermelon); condoms lubricated with mild anesthetic to delay ejaculation; extra-large, anatomically shaped condoms; and condoms lubricated to create either a cool sensation or an extra warm sensation, among others. See the Figure for a sampling of brands. The original (although now improved) Prudence brand still sells briskly at 27¢ per piece, well below the affordability guideline.

Cross-subsidization involves introducing new brands at higher prices while still maintaining at least 1 brand that meets affordability guidelines.

**FIGURE:**
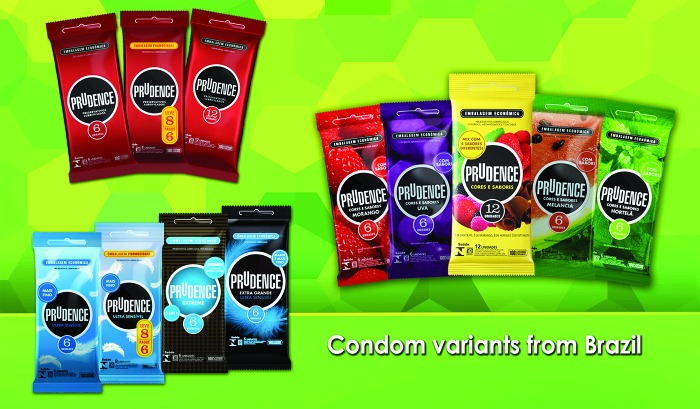
Sample of DKT International’s Prudence Condom Brand Varieties Sold in Brazil

For the past decade, DKT/B has become a solid commercial operation that fulfills its basic social function but makes most of its decisions on the basis of profits. By the end of 2001, DKT had invested nearly $6 million in the operation; while we never expected to see these funds returned, it now appears that they may be. The first profit remittance to DKT’s Washington office was $30,000 in 2004. Since then, a total of $3.9 million from the Brazil project has been returned to headquarters. These funds were used primarily to start new programs in Ghana and Mozambique, programs that include the social marketing of contraceptives, a small number of franchise clinics, and sale of medical abortion drugs. DKT/B’s after-tax profits and remittances are shown in Table 2.

Profits from DKT country programs like Brazil’s help start new programs in poorer countries like Ghana and Mozambique.

**TABLE 2:** DKT do Brasil: Revenue, Profits, and Remittances to Washington, DC, Headquarters, 2000–2013 (millions, US dollars)

Year	Revenue	Profit After Tax	Remittances to DC Headquarters	Cumulative Remittances
2000	5.9	-1.9	–	–
2001	5.7	-0.7	–	–
2002	4.8	-1.7	–	–
2003	3.9	-1.6	–	–
2004	4.7	0.04	0.03	0.03
2005	6.5	0.9	0.12	0.15
2006	8.1	0.7	0.16	0.31
2007	10.8	0.7	0.26	0.57
2008	13.3	0.5	0.26	0.83
2009	11.5	0.9	0.40	1.23
2010	14.8	0.8	0.60	1.83
2011	20.0	1.3	0.60	2.43
2012	21.5	1.3	0.80	3.23
2013	22.1	0.6	0.65	3.88

In pure business terms, these remittances would not constitute a good return on investment, even if all distributions continue on schedule. Too much money, too many years to provide an investment return. But the objective of a social marketing project is to achieve a social benefit, and that has been achieved. The project, by importing and marketing low-cost condoms, brought down the price of other brands as well as introducing a low-cost condom of its own. DKT/B had joined and helped precipitate a dramatic increase in condom sales and use throughout Brazil. DKT’s share in the commercial market grew from a very small fraction in the early 1990s to well over 20% by 2011 (Table 1). While it is not possible to calculate the health impact of these sales with precision, the impact on HIV and pregnancy prevention was significant. The 458 million DKT condoms sold during the decade between 1995 and 2004, for example, prevented an estimated 70,000 HIV infections and 1.2 million unwanted pregnancies. (HIV estimates derived from Family Health International’s AVERT model^8^ and estimate of unwanted pregnancies prevented from Marie Stopes International’s Impact Calculator.^9^)

Further, a great many family planning and HIV-prevention projects produce no financial returns at all. So DKT/B has become a different type of creature. It is a Brazilian business, part of Brazil’s normal economic landscape, and, at the same time, an integral part of an international family planning organization and a contributor to that organization’s charitable goals.

## MORALE ISSUES?

DKT’s leadership was originally concerned that its profit-making programs would be resented—or at least envied—by program directors in the lowest-income countries where financial self-sufficiency is not possible. But there has always been an important trade-off. DKT’s programs in the Democratic Republic of the Congo and Mozambique, for example, are serving the clients with the greatest need because those are among the poorest populations in the world and contraceptive prevalence is extremely low. Virtually every contraceptive provided or sold in those environments makes an important difference, so everyone has something to brag about.

And, even in the poorest countries, DKT programs do not rely entirely on donor support to function and expand. Cross-subsidization always chips in. The program in Ethiopia, for example, generated $3.4 million in revenue in 2013. While this was only 18% of total program expenses that year, the contribution makes a substantial difference and helps provide a good cost-benefit ratio for DKT’s donors. Worldwide, DKT programs generated $94 million in sales revenue in 2013 against total program expenses of $131 million. The balance is made up with donor funds.

## AN UNINTENTIONAL DESIGN

DKT International has evolved into something its founders never anticipated: an international nonprofit family planning organization with 3 distinctly different components:

- Contraceptive marketing programs in middle-income countries like Brazil, Egypt, Indonesia, Mexico, and the Philippines, where annual per-capita GNI exceeds $9,000. These programs have—or will—produce true operating profits from which remittances are made to DKT headquarters to support DKT’s subsidized programs in low-income countries (significant remittances to date from Brazil, Indonesia, and the Philippines).
- Subsidized social marketing and other family planning programs in low-income (PPP-adjusted GNI <$2,000) countries where cross-subsidization covers part of program expenses but the lion’s share is paid by donors.
- Programs in the economically in-between countries where progress toward the “A” category may well be possible as economic growth continues.

## LESSONS LEARNED

While DKT’s structure evolved without an originating design, there is a good deal to be learned, both from the Brazil program experience and from DKT’s progression as it evolved from a traditional family planning social marketing NGO in the 1990s to the 3-tiered organization described above. Three factors were central to this process:

1. As noted, rising incomes in Asian and Latin American countries have made it possible to charge higher prices for contraceptives without exceeding affordability guidelines.
2. DKT headquarters recognized and supported sales revenue generation as a policy. We introduced a generous scale of country-director commissions that rewarded not only increases in CYPs each year, but also fund-raising revenue and, importantly, revenue from sales. Commissions continue to be paid for all three.
3. When recruiting new talent, we increasingly looked for candidates with commercial sales experience to head up DKT’s overseas programs.

Because of these and related factors, DKT gradually took on an organizational culture in which steps toward profitability became positive goals recognized by everyone. Many of our new country directors, for example, found it unnatural to sell products below cost (“Why would you do that?”) as is traditionally done in highly subsidized social marketing programs (including DKT’s).

While there is a continuous discussion in DKT about whether to prioritize CYPs or income, the two are usually not in conflict. With the right pricing structure, as CYPs go up, so does revenue, although everyone understands that price increases must be gradual and carefully tested, and that at least 1 brand must remain within the affordability guidelines.

## OTHER APPROACHES

There are of course other approaches for increasing social marketing program revenue. Some Latin American programs, for example, have raised revenue by selling a range of health and hygiene products,^10^ and one or two programs have provided clinic and lab services at a profit as part of what they offer. While these approaches have enjoyed modest success in particular environments, they have not made a significant contribution to contraceptive social marketing revenue worldwide. That revenue, which now exceeds $150 million per year, results overwhelmingly from the sale of contraceptives.

Sales of other health and hygiene products have enjoyed modest success in certain places but none like the sale of contraceptives.

DKT’s own experience bears this out. Lubricants, for example, are considered highly appropriate in DKT’s and many other programs, especially in conjunction with condom sales, and pregnancy test strips are also sold in several DKT programs. But these products seldom have good margins, and they do not contribute significantly to profits. Neither do such products as sanitary napkins and razor blades, which have also been tried. Overwhelmingly it is birth control products—condoms, oral contraceptives, emergency contraceptive pills, and medical abortion drugs—that produce solid income streams and good margins.

For those who wish to try the paths towards self-sufficiency described in this paper, the basic formula is fairly simple: Create a culture where increasing sales revenue and profitability are seen as important virtues. Hire people who believe in this principle, and reward them for following it.

## Footnotes

* The value of 100 condoms per year differs from the USAID factor of 120.

## Notes

**Peer Reviewed**
